# HIV Infection among Young People in Northwest Tanzania: The Role of Biological, Behavioural and Socio-Demographic Risk Factors

**DOI:** 10.1371/journal.pone.0066287

**Published:** 2013-06-21

**Authors:** Francesca Lemme, Aoife M. Doyle, John Changalucha, Aura Andreasen, Kathy Baisley, Kaballa Maganja, Deborah Watson-Jones, Saidi Kapiga, Richard J. Hayes, David A. Ross

**Affiliations:** 1 London School of Hygiene and Tropical Medicine, London, United Kingdom; 2 Mwanza Intervention Trials Unit, National Institute for Medical Research, Mwanza, Tanzania; 3 National Institute for Medical Research, Mwanza, Tanzania; Rollins School of Public Health, Emory University, United States of America

## Abstract

**Background:**

Young people are at high risk of HIV and developing appropriate prevention programmes requires an understanding of the risk factors for HIV in this age group. We investigated factors associated with HIV among participants aged 15–30 years in a 2007–8 cross-sectional survey nested within a community-randomised trial of the MEMA kwa Vijana intervention in 20 rural communities in northwest Tanzania.

**Methods:**

We analysed data for 7259(53%) males and 6476(47%) females. Using a proximate-determinant conceptual framework and conditional logistic regression, we obtained sex-specific Odds Ratios (ORs) for the association of HIV infection with socio-demographic, knowledge, behavioural and biological factors.

**Results:**

HSV-2 infection was strongly associated with HIV infection (females: adjOR 4.4, 95%CI 3.2–6.1; males: adjOR 4.2, 95%CI 2.8–6.2). Several socio-demographic factors (such as age, marital status and mobility), behavioural factors (condom use, number and type of sexual partnerships) and biological factors (blood transfusion, lifetime pregnancies, genital ulcers, *Neisseria gonorrhoeae*) were also associated with HIV infection. Among females, lifetime sexual partners (linear trend, p<0.001), ≥2 partners in the past year (adjOR 2.0, 95%CI 1.4–2.8), ≥2 new partners in the past year (adjOR 1.9 95%CI 1.2, 3.3) and concurrent partners in the past year (adjOR 1.6 95%CI 1.1, 2.4) were all associated with HIV infection.

**Conclusions:**

Efforts must be intensified to find effective interventions to reduce HSV-2. Effective behavioural interventions focusing on reducing the number of sexual partnerships and risk behaviour within partnerships are also needed. An increase in risky sexual behaviour may occur following marriage dissolution or when a young woman travels outside of her community and interventions addressing the needs of these subgroups of vulnerable women may be important.

**Trial Registration:**

ClinicalTrial.gov NCT00248469.

## Introduction

Young people aged 15–24 years account for an estimated 40% of new adult HIV infections worldwide [1]. The United Nations General Assembly Special Session on HIV/AIDS (UNGASS) *Declaration of Commitment* established a target to reduce HIV prevalence by 25% in young people aged 15–24 in the most affected countries by 2005 [2]. HIV prevalence has declined among young people since 2000–2001 in 16 of the 21 high prevalence countries with sufficient data, including Tanzania. However, significant declines of 25% were seen in only ten of these countries [3]. Effective HIV prevention in young people therefore remains an urgent priority, but the design of HIV prevention interventions requires a clear understanding of the social, behavioural and biological factors that influence the risk of HIV infection.

Previous studies in Tanzania and elsewhere have identified a number of factors that are commonly associated with increased risk of HIV infection such as age [4], [5], [6], socio-economic status [4], [7], [8], educational status [9], [10]; marital status [4], [6], [9], [10], mobility [6], [11], high risk sexual behaviours [4], [5], [6], [10] and co-infection with other sexually transmitted infections (STIs) [6], [9], [12], [13], [14]. However, the relative contribution of these factors varies between locations and also can vary over time as the epidemic matures and prevention efforts intensify [7], [8]. The majority of these studies were carried out in the late 1990’s and early 2000’s and few focused specifically on young people. Between 1998 and 2008 a community-randomised controlled trial was conducted in rural Tanzania to evaluate the impact of the MEMA kwa Vijana (“Good things for young people”) adolescent sexual and reproductive health intervention on biological, behavioural, attitudinal and knowledge outcomes. Ten of 20 randomly chosen communities received the intervention. The aim of the intervention was to reduce incidence of HIV, other STIs and unintended pregnancies by providing young people with the knowledge and skills to enable them to delay sexual debut and reduce sexual risk taking. The design of the trial [15] and intervention [16] are described elsewhere. Impact evaluations conducted three years (2001/2) and nine years (2007/8) post-intervention demonstrated that the MEMA kwa Vijana intervention led to an improvement in young people’s sexual and reproductive health knowledge and reported attitudes and some reported sexual behaviours. However, no impact was seen on the prevalence of HIV and other STIs [17], [18].

We used data from the more recent 2007/8 survey to investigate risk factors for HIV among young people living in rural Tanzania, and to identify and prioritise target areas for future interventions.

## Materials and Methods

### Study Design

Between June 2007 and July 2008, approximately nine years after the introduction of the MEMA kwa Vijana intervention, a cross-sectional survey was conducted in the 20 trial communities to evaluate the long-term impact of the intervention on the sexual and reproductive health of young people. Participants had attended at least one of school years 5–7 in the trial schools between 1999 and 2002.

Details of the design of the survey, selection of study participants and inclusion/exclusion criteria can be found elsewhere [18], [19].

Attendees were interviewed about their knowledge, reported attitudes and reported sexual behaviours, and were asked to provide urine and venepuncture blood specimens. In addition, HIV counseling and testing was offered using parallel HIV rapid tests on a fingerprick blood specimen (SD Bioline HIV-1/2 3.0 (Standard Diagnostics Inc) and Determine HIV1/2 (Abbott Laboratories)). A clinician asked about STI symptoms (males and females) and examined males for signs of STIs. This analysis is restricted to the 13,735 participants (99.4%) who provided serum and urine samples for STI testing.

Each of the sexual knowledge and attitudes scores was based on three questions (detailed elsewhere [18]). Only participants correctly answering all three questions were deemed to have good sexual knowledge or attitudes.

Multiple answers were allowed for the question on occupation. The majority of participants (77%) had one occupation only at the time of interview, but 19% reported two jobs and 4% reported three or more. For the statistical analyses reported here, separately for males and females, multiple occupations were classified into the occupation with the lowest frequency. For example, the majority of participants were farmers, so those who were farmers but also had another occupation were classified into the other occupation.

### Laboratory Methods

Sera were tested for HIV in parallel, using 3^rd^ generation Murex HIV 1.2.O ELISA (Abbott-Murex, Dartford, UK) and 3^rd^ generation Vironostika HIV Uniform II plus O (Biomeriux, Boxtel, Netherlands). Sera with discordant or indeterminate ELISA results were retested up to two more times on both ELISAs. Persistently discordant samples were tested for P24 antigen using Biorad Genetic System HIV1 Ag EIA (Biorad, Lacoquette, France) and P24 negative samples were tested with Inno-Lia HIV1/2 score Assay (Inno-Genetics NV, Gent, Belgium). Inno-Lia negative and indeterminate specimens were classified as negative.

Sera were tested for antibodies to herpes simplex virus type 2 (HSV2) using type-specific Kalon IgG ELISA (Kalon Biologicals, Guildford, UK) according to manufacturers instructions. Persistently indeterminate samples were classified as negative.

Serological tests for syphilis were conducted and urine specimens were tested for *Chlamydia trachomatis* (CT) and *Neisseria gonorrhoeae* (NG) by PCR. Details of laboratory methods can be found elsewhere [18].

### Statistical Methods

We analysed associations with HIV infection using a proximate determinants conceptual framework [20]. Exposures were grouped into the following four families based on their hypothesised proximity to the outcome:

1. Socio-demographic: age, ethnic group, religion, educational level, occupation, marital status, length of time slept away from their community during the past year
2. Sexual knowledge and attitudes: knowledge of HIV, knowledge of sexually transmitted diseases (STDs), reported sexual attitudes
3. Sexual and reproductive behaviours: age at first sex, condom use (ever and during past year), modern contraception (condom, oral contraceptive pill, injectable hormonal contraception use), number of partners (lifetime and during past year), lifetime number of pregnancies (females only), number of new partners and concurrent partnerships during past year.
4. Biological: blood transfusion during the past 5 years, reported number of injections during the past year, whether circumcised (males only - based on clinical examination) and timing of circumcision, reported abnormal genital discharge or genital ulcers during the past year, seropositive for HSV2 or syphilis, infected with *Chlamydia trachomatis*, or *Neisseria gonorrhoeae.*

Data were analysed separately for males and females. Conditional logistic regression was used to obtain odds ratios (ORs) for HIV infection, conditioned on study community to take into account correlations induced by the clustering of participants within geographically-defined communities and potential confounding due to variation in underlying rates of HIV and other STIs between communities. Intervention status was not considered, as this showed no significant effect on the prevalence of HIV infection [18].

Likelihood ratio tests were used at each step of the analysis. Age was considered an *a priori* potential confounder and included in all models.

Association of each socio-demographic factor with HIV, adjusted by age, was firstly investigated. Socio-demographic factors with p<0.20 were included in a multivariate “socio-demographic” model and were then retained in a core model if the adjusted p-value was<0.10. Next, knowledge and attitude factors were added to this core model one by one. Those with a p-value<0.20 were included in a multivariate “knowledge and attitude” model and were retained in the model if the adjusted p-value was<0.10. Associations with behavioural and biological factors were determined in a similar way. This strategy allowed us to assess the effect of variables at each level of the framework, adjusted for the more distal variables.

### Ethics

The trial protocol received ethical and research clearance from the Tanzanian Medical Research Coordinating Committee and the Ethics Committee of the London School of Hygiene and Tropical Medicine. Signed informed consent was obtained from each participant on the day of the survey. Additional written consent was obtained from parents for participants under the age of 18 years.

## Results

Data were analysed for 7259 (53%) males and 6476 (47%) females.

The median age was 23 years for males and 22 years for females. The majority of participants were members of the Sukuma tribe (males: 78%, females: 82%), were Christian (males: 81%, females: 89%), were not educated beyond primary school (males: 79%, females: 88%) and worked as farmers (males: 46%, females: 64%) (Table 1). The majority of males had never been married (63%) while the majority of females were married or cohabiting with a partner (56%).

**Table 1:** Risk factors for HIV infection: odds ratios (ORs) for selected socio-demographic factors.

		Males (N = 7259)			Females (N = 6476)		
Exposures	Categories	Number	Prevalence n (%)	Adjusted OR^b^ (95% CI)	Number	Prevalence n (%)	Adjusted OR^c^ (95% CI)
Age	<21 years	2039	10 (0.5%)	1 pt<0.001	2623	66(2.5%)	1 pt<0.001
	21–22 years	1966	26 (1.3%)	1.87(0.85–4.12)	1855	85(4.6%)	1.61(1.14–2.26)
	23–24 years	1903	43 (2.3%)	2.69(1.25–5.79)	1490	81(5.4%)	1.94(1.36–2.77)
	> = 25 years	1350	54 (4.0%)	4.04(1.86–8.78)	506	20(5.9%)	2.11(1.31–3.38)
Ethnic group	Non Sukuma	1567	28 (1.8%)	1 p = 0.77	1196	47(3.9%)	1 p = 0.65
	Sukuma	5684	105 (1.9%)	1.08(0.65–1.78)	5274	215(4.1%)	1.09(0.74–1.60)
Religion	Christian	5850	106 (1.8%)	1 p = 0.43	5733	229(4.0%)	1 p = 0.003
	Moslem	326	5 (1.5%)	0.75(0.27–2.08)	274	20(7.3%)	2.21(1.34–3.65)
	None/other	1076	21(2.0%)	0.74(0.45–1.22)	453	11(2.4%)	0.59(0.31–1.12)
Education	Primary or less	5723	121 (2.1%)	1 p = 0.24^d^	5592	237(4.2%)	1 p = 0.22^d^
	Secondary or higher	1529	12 (0.8%)	0.69(0.36–1.31)	874	25(2.9%)	0.75(0.48–1.19)
Occupation	Domestic/Farmer^a^	3312	72(2.2%)	1 p = 0.006	4419	186(4.2%)	1 p<0.001
	At school/university	1732	5(0.3%)	0.29(0.11–0.76)	806	6(0.8%)	0.19(0.08–0.46)
	Business	1576	43(2.7%)	1.35(0.90–2.02)	418	23(5.5%)	1.24(0.77–1.97)
	None/Other	530	10(1.9%)	0.90(0.46–1.78)	803	45(5.6%)	1.23(0.85–1.77)
Marital status	Married	2435	77(3.2%)	1 p = 0.034	3644	130(3.6%)	1 p<0.001
	Widowed/Separated/Divorced	226	10(4.4%)	1.59(0.80–3.17)	622	56(9.0%)	2.57(1.84–3.61)
	Never married	4598	46(1.0%)	0.65(0.43–1.00)	2210	76(3.4%)	1.48(1.07–2.05)
Time slept away	Never	2340	40(1.7%)	1 pt = 0.20	3381	136(4.0%)	1 pt = 0.055
past year	Up to 1 month	3317	58(1.8%)	1.02(0.67–1.55)	2140	71(3.4%)	0.82(0.61–1.11)
	1 month to 3 months	721	16(2.2%)	1.31(0.69–2.47)	536	24(4.5%)	1.12(0.71–1.78)
	>3 months	806	16(2.0%)	1.47(0.79–2.73)	422	31(7.4%)	1.79(1.17–2.74)

NOTE: OR: odds ratio; CI: confidence interval; p: pvalue from likelihood-ratio test; pt: p value from likelihood-ratio test for linear trend; NA: not applicable.

a The category Domestic/Farmer includes females who reported being either housewives or farmers and males who reported being farmers.

b *Adjusted for age, occupation and marital status.*

c *Adjusted for age, religion, occupation, marital status and length of time slept away past year.*

d *for males adjusted for age and marital status; for females adjusted for age, religion, marital status and length of time slept away past year.*

HIV prevalence was 1.8% in males and 4.1% in females. HSV2 prevalence was 25.8% in males and 41.4% in females.

Only 50% of males and 34% of females were classified as having good knowledge of sexually transmitted diseases (STDs), while 70% of males and 64% of females were classified as having good knowledge of HIV (Table 2).

**Table 2:** Risk factors for HIV infection: odds ratios (ORs) for selected sexual knowledge and reported behaviours.

		Males (N = 7259)			Females (N = 6476)		
Exposures	Categories	Number	Prevalencen (%)	Adjusted OR(95% CI)	Number	Prevalencen (%)	Adjusted OR(95% CI)
Knowledge of HIV	0–2answers correct	2202	47 (2.1%)	1 p = 0.20^a^	2297	74(3.2%)	1 p = 0.049^b^
	3 answers correct	5040	86 (1.7%)	0.78(0.54–1.14)	4159	188(4.5%)	1.33(1.00–1.77)
Knowledge of STD	0–2 answers correct	3610	69 (1.9%)	1 p = 0.87^a^	4245	171(4.0%)	1 p = 0.99^b^
	3 answers correct	3629	64 (1.8%)	1.03(0.72–1.47)	2213	91(4.1%)	1.00 (0.76–1.31)
Sexual Attitudes	0–2 answers correct	5447	102 (1.9%)	1 p = 0.87^a^	5781	238(4.1%)	1 p = 0.63^b^
	3 answers correct	1805	31 (1.7%)	1.04(0.68–1.58)	685	24(3.5%)	0.90(0.58–1.40)
Age at first sex	> = 16 years	4669	91(2.0%)	1 p = 0.71 c	4244	165(3.9%)	1 p = 0.105^e^
	<16 years	1902	39(2.0%)	1.08(0.73–1.60)	1762	90(5.1%)	1.27(0.95–1.69)
Ever used condom	No	3079	40(1.3%)	1 p = 0.25 c	3684	113(3.1%)	1 p = 0.037^e^
	Yes	4180	93(2.2%)	1.26(0.85–1.88)	2791	149(5.3%)	1.36(1.02–1.81)
No. lifetime partners	0	657	1(0.2%)	NA	449	6(1.3%)	0.49(0.20–1.22)
	1–2 (male), 1 (female)	1783	14(0.8%)	0.44(0.25–0.80)	1820	39(2.1%)	0.46(0.31–0.67)
	3–4 (male), 2 (female)	1861	32(1.7%)	0.75(0.49–1.14)	1908	69(3.6%)	0.66(0.49–0.90)
	≥5 (male), ≥3 (female)	2926	86(2.9%)	1 pt = 0.003 c	2281	148(6.5%)	1 pt<0.001^e^
No. partners past year	0–1	4174	57(1.4%)	1 p = 0.10^a^	5820	204(3.5%)	1 p<0.001^d^
	> = 2	3085	76(2.5%)	1.36(0.95–1.96)	656	58(8.8%)	1.96(1.39–2.76)
No. new partners overlast year	0	2269	36(1.6%)	1 pt = 0.11^a^	4704	168(3.6%)	1 pt = 0.027^d^
	1	2053	45(2.2%)	1.69(1.07–2.67)	1182	57(4.8%)	1.18(0.84–1.66)
	> = 2	2077	44(2.1%)	1.47(0.91–2.38)	206	21(10.2%)	1.94(1.15–3.30)
>1 partner at same timepast year	No	5048	73(1.5%)	1 p = 0.20^a^	6050	229(3.8%)	1 p = 0.032^d^
	Yes	2211	60(2.7%)	1.27(0.88–1.82)	426	33(7.8%)	1.58(1.06–2.37)

NOTE: OR: odds ratio; CI: confidence interval; STD: sexually transmitted diseases; p: pvalue from likelihood-ratio test; pt: pvalue from likelihood -ratio test for linear trend; NA: not applicable.

a *Adjusted for age, occupation and marital status.*

b *Adjusted for age, religion, occupation, marital status and length of time slept away past year.*

c *Adjusted for age, occupation, marital status and number of lifetime partners Adjusted for age, religion, occupation, marital status, length of time slept away past year, HIV knowledge and ever used condom.*

e *Adjusted for age, religion, occupation, marital status, length of time slept away past year, HIV knowledge, ever used condom and number of lifetime partners.*

### Socio-demographic Factors (Table 1)

Following adjustment for other statistically significant socio-demographic factors, the odds of HIV infection increased with age for both sexes.

While for males religion was not significantly associated with HIV infection, Moslem female participants had more than double the odds of HIV compared to Christians (adjOR 2.21, 95%CI 1.34–3.65) (Table 1).

Students had a much lower odds of HIV compared to farmers and domestic workers (males: adjOR 0.29, 95%CI 0.11–0.76; females: adjOR 0.19, 95%CI 0.08–0.46).

There was strong evidence of an association between HIV risk and marital status among women. Women who were widowed, separated or divorced had more than twice the odds of HIV (adjOR 2.57, 95%CI 1.84–3.61) compared to married females, and risk was also higher among never married women (adjOR 1.48, 95%CI 1.07–2.05). The association was weaker among men and never married men had a lower risk of HIV when compared to married men (adjOR 0.65, 95%CI 0.43–1.00).

The majority of females had not slept outside their community during the past year (52%), while most males had spent time away (67%). While there was no significant association between HIV prevalence and time spent away in males, there was some evidence of a trend in females (p-trend = 0.055). HIV prevalence among females who were away from their community for more than three months was much higher than among less mobile females (7% versus 4%), and this association remained after adjustment for other socio-demographic factors (adjOR 1.79, 95%CI 1.17–2.74).

Although secondary or above education was associated with lower HIV prevalence in the unadjusted analysis, that association was no longer significant after adjusting for age among females, or age and marital status among males.

After adjusting for more proximal knowledge, behaviour and biological factors, age was the only factor that remained significantly associated with HIV among males (data not shown). Among females, after adjusting for more proximal factors, the association with all socio-demographic factors remained, except for time away from the community.

### Sexual Knowledge, Reported Attitudes and Reported Behaviours (Table 2)

After adjustment for statistically significant socio-demographic factors, we found weak evidence that female participants with a good knowledge of HIV had a higher risk of the infection (adjOR = 1.33; 95%CI 1.00–1.77).

About half of both male and female participants reported ever having used a condom. Females who reported ever having used a condom had a higher risk of HIV compared to those who had not (adjOR 1.36, 95%CI 1.02–1.81).

About half of sexually active male participants (48%) reported at least five lifetime sexual partners, whereas a slightly lower proportion of female participants (38%) reported at least three lifetime sexual partners. As expected, the odds of HIV infection increased with increasing number of sexual partners. When compared to males, a much lower proportion of females reported more than one sexual partner or at least one new sexual partner during the past year, or having had concurrent sexual partnerships during the past year. Among males, there was no evidence that HIV prevalence differed according to these recent partnership patterns. On the other hand, there was strong evidence to suggest that the small proportion of females who reported at least two sexual partners in the past year had higher risk of HIV (adjOR 1.96, 95%CI 1.39–2.75). There was also evidence that HIV risk was increased among females who reported a larger number of new partners over the past year or who had engaged in concurrent partnerships. There was no evidence of an association between HIV status and recent condom use or lifetime or recent use of hormonal contraception (data not shown).

Among males, after adjusting for more proximal biological factors, the association of increasing lifetime partners with HIV still remained. Among females, after adjusting for behavioural and biological factors, the association with increasing lifetime partners and partners in the past year remained (data not shown).

### Biological Factors (Table 3)

Very few participants reported having had a blood transfusion during the five years prior to the survey. Among those who did, none of the males and 16 of the females (10%) were HIV positive, and, despite the small numbers, there was some evidence of an increase in HIV risk among females who reported a transfusion in the past five years (adjOR 2.02, 95%CI 1.11–3.67). There was no evidence that having had at least one injection in the past year was associated with HIV, either in females (adjOR 1.10 95%CI 0.84–1.44) or in males (adjOR 0.71, 95%CI 0.44–1.15).

**Table 3:** Risk factors for HIV infection: odds ratios (ORs) for selected biological factors.

		Males (N = 7259)			Females (N = 6476)		
Exposures	Categories	Number	Prevalencen (%)	Adjusted OR^a^(95% CI)	Number	Prevalencen (%)	Adjusted OR^b^(95% CI)
Had blood transfusionpast 5 yrs	No	7192	133(1.9%)	NA	6304	246(3.9%)	1 p = 0.030
	Yes	57	0(0.0%)		161	16(9.9%)	2.02(1.11–3.67)
Number of injectionspast year	0	5617	108(1.9%)	1 p = 0.15	3502	132(3.8%)	1 p = 0.46
	> = 1	1598	23(1.4%)	0.71(0.44–1.15)	2886	128(4.4%)	1.10(0.84–1.44)
Circumcised (clinically observed)	No	4248	85(2.0%)	1 p = 0.69	–	–	–
	Yes, aftersexual debut	1048	23(2.2%)	1.04(0.61–1.75)	–	–	–
	Yes, beforesexual debut	1346	12(0.9%)	0.76(0.37–1.52)	–	–	–
No. lifetime pregnancies	0–1	–	–	–	5264	219(4.2%)	1 p = 0.014
	> = 2	–	–	–	1187	43(3.6%)	0.63(0.43–0.92)
Reported abnormal genital	No	6643	112(1.7%)	1 p = 0.95	6159	241(3.9%)	1 p = 0.72
discharge during past year	Yes	606	21(3.5%)	1.02(0.59–1.76)	300	21(7.0%)	1.10(0.65–1.87)
Reported genital ulcers during	No	6813	111 (1.6%)	1 p = 0.042	6099	229(3.8%)	1 p<0.001
past year	Yes	434	22 (5.1%)	1.72(1.04–2.83)	364	33(9.1%)	2.14(1.42–3.22)
HSV2	Negative	5383	43(0.8%)	1 p<0.001	3794	54(1.4%)	1 p<0.001
	Positive	1876	90(4.8%)	4.18(2.84–6.15)	2682	208(7.8%)	4.42(3.20–6.12)
Active Syphilis (*TPPA+, RPR+*)	Negative	6858	115(1.7%)	1 p = 0.16	6029	233(3.8%)	1 p = 0.51
	Positive	257	15(5.8%)	1.55(0.86–2.80)	314	21(6.7%)	1.18(0.73–1.91)
Lifetime Syphilis (*TPPA+*)	Negative	6858	115(1.7%)	1 p = 0.34	6029	236(3.9%)	1 p = 0.40
	Positive	401	18(4.5%)	1.31(0.76–2.24)	447	29(6.5%)	1.20(0.79–1.83)
Chlamydia trachomatis	Negative	7104	127(1.8%)	1 p = 0.43	6319	253(4.0%)	1 p = 0.63
	Positive	152	6(4.0%)	1.45(0.60–3.50)	153	9(5.9%)	1.20(0.58–2.46)
Neisseria Gonorrhoeae	Negative	7228	129(1.8%)	1 p = 0.011	6449	259(4.0%)	1 p = 0.16
	Positive	28	4(14.3%)	5.76(1.81–18.26)	23	3(13.0%)	2.77(0.77–9.96)

NOTE: OR: odds ratio; CI: confidence interval; HSV2: herpes simplex virus 2; p: pvalue from likelihood-ratio test; pt: pvalue from likelihood -ratio test for linear trend; NA: not applicable.

a *Adjusted for age, occupation, marital status, number of lifetime partners, reported genital ulcers during past year, HSV-2 infection, Neisseria gonorrhoea.*

b *Adjusted for age, religion, occupation, marital status, length of time slept away past year, HIV knowledge, ever used condom, number of lifetime partners, number of lifetime pregnancies, ever had blood transfusion past 5 yrs, reported genital ulcers during past year, HSV2 infection.*

Less than half of males (36%) were circumcised. HIV prevalence among circumcised and uncircumcised males was similar (approximately 2%), while HIV prevalence among those circumcised before sexual debut was much lower (0.9%). After adjustment for distal factors and other significant biological factors, there was no evidence of an association between circumcision before sexual debut and HIV infection (adjOR 0.76, 95%CI 0.37–1.52).

One fifth of women had had at least two lifetime pregnancies, and these women had a lower risk of HIV (adjOR 0.63, 95%CI 0.43–0.92).

For both sexes, there was no evidence of an association between reported abnormal genital discharge during the past 12 months and HIV-infection. Approximately half of HSV2-infected participants reported genital ulcers during the 12 months prior to the survey (54.4% females; 47.5% males). After adjusting for all other significant factors, there was strong evidence of an association between reported genital ulcers in the 12 months prior to the survey and HIV infection in females (adjOR 2.14, 95%CI 1.42–3.22) and weaker evidence of an association in males (adjOR 1.72, 95%CI 1.04–2.83).

As expected, there was very strong evidence of an association between HSV2 infection and HIV infection (females: adjOR 4.42, 95%CI 3.20–6.12; males: adjOR 4.18, 95%CI 2.84–6.15). HIV prevalence was also much higher among participants who tested positive for other STIs, such as active or lifetime syphilis, *Chlamydia trachomatis or Neisseria gonorrhoeae*. After adjusting for all relevant socio-demographic, sexual knowledge, behavioural and biological factors, there was no evidence of an increased risk of HIV-infection among participants who tested positive for syphilis or *Chlamydia trachomatis*. However, males with *Neisseria gonorrhoeae* had a much higher risk of HIV (adjOR 5.76, 95%CI 1.81–18.26).

## Discussion

We identified several socio-demographic, behavioural and biological factors that were associated with HIV infection in this population of young people living in a rural area of northwest Tanzania in 2007/08. It is sobering to note that, despite almost ten years of intervention in these communities, HIV prevalence has remained high, and the factors associated with HIV infection in our study did not differ greatly from risk factors identified in studies conducted in Mwanza 10–15 years ago [4], [6], [10].

As expected, one of the strongest associations in this study was between HIV and HSV-2 infection. The prevalence of HSV-2 was also very high (26% in males and 41% in females), indicating that HSV-2 remains a major public health problem in this population. After adjustment for all relevant factors, there was also a highly significant association between genital ulcers and HIV-infection for both sexes. The association between HSV-2 and HIV is well established [13], [14], [21], [22], [23], and recent HSV-2 pathogenesis research suggests that there are a number of biological mechanisms through which HSV-2 increases HIV incidence, many of which occur in the absence of active ulcers [24]. Clinical trials of HSV-2 suppressive therapy in Mwanza and elsewhere have not demonstrated a reduction in HIV transmission [25], [26], [27], [28]. Work continues to develop more efficacious suppressive therapy regimens, HSV vaccines, and to explore the potential protective effects of Tenofovir gel [24], but in the meantime encouraging lower-risk sexual activity and condom use remain the only effective preventive measures available. Among males, infection with Neisseria Gonorrhoeae was also associated with HIV infection. It is important, therefore, that efforts are increased to educate community members about the association between HIV and STIs, and to encourage them to seek treatment promptly when they suspect that they might have an STI.

One unexpected finding was that Moslem women were at higher risk of HIV-infection than Christian women. One explanation might be that Moslems are more likely to live in areas of higher HIV prevalence within study communities such as roadside settlements and urban areas [10]. Although we conditioned our analysis on community, there was considerable rural/urban variation between the villages within the same community. It is also possible that Moslem women had less access to health promotion information, were less able to negotiate condom use, and/or were more likely to be in a polygamous relationship. Extensive qualitative work carried out among young people in these study communities has highlighted the existence of restrictive norms on young people’s sexual activity, norms which are associated with a secrecy among sexually active young people that sometimes results in increased sexual risk [29]. There is some evidence to suggest that pressure to maintain sexual respectability was greater for those from devout Christian or Muslim families [29], and this may partly explain our findings. It will be important to engage with the Moslem community to try to understand if and why young females in their community are at higher risk of HIV.

Quantitative and qualitative data from this region and elsewhere suggest that young people’s sexual behaviour is influenced by broader social determinants such as culture, economics and community social and geographical characteristics [30], [31], [32]. Given the prevailing social norms regarding young people’s sexual activity, there is increasing recognition of the importance of engaging the whole community, including parents, teachers, religious and local leaders, in order to prevent HIV among young people [33], [34]. It is likely that broader and more rapid reductions in sexual risk behaviour and hence HIV may only be possible when social norms are changed to allow young people to practice safer sex, and evaluation of interventions with parents and other community groups is warranted.

An important finding of this study was that being in full-time education was protective for both sexes. Studies in Mwanza earlier in the epidemic found that higher education was associated with an increased risk of HIV [4], [10]. The findings from this study are in line with more recent national data from Tanzania which suggests that risk of HIV is now stable among those with no education and decreasing among those with secondary education or higher [35], [36]. Selection of our study population was based on their attendance at an intervention or comparison primary school and hence all had reached at least the 5^th^ year/standard with most having completed the full seven years of primary school and few progressing on to education beyond secondary school. They represented therefore the ‘middle’ of the spectrum of educational level and an association with HIV may only be seen if those with little or no education and/or with high levels of education are included in the analysis. Current students in our study reported lower levels of sexual risk behavior (data not shown) which is consistent with the lower prevalences of HIV and HSV2 observed in this group. Efforts to encourage young people, especially young girls, to remain in full-time education have been successful in some settings [37] and other similar studies are ongoing. However, in the short-term it is unlikely that secondary school attendance will increase dramatically in these rural areas. It will be important to get a better understanding of what factors encourage secondary school students to practice safer sex, for example, improved knowledge, increased access to resources, parental support and/or supervision, improved aspirations for the future [29]. It may then be possible to design interventions to motivate both school-going and non-school going young people to protect themselves while also working in the community to create an enabling environment for them to do so.

The higher odds of infection that was observed among those who were separated, divorced or widowed has been seen consistently in the past [4], [6], [10]. Reverse causality is one possible explanation as HIV can lead to the dissolution of a marriage, due to death of the partner from HIV or because the husband no longer wants to remain in the relationship if his partner’s positive HIV status becomes known or if she becomes ill or is infertile. However, the increased risk among non-married and separated, widowed and divorced young females may be due to an increase in the number or variation in the type of sexual partners. We need to closely examine the factors that lead to an increased risk of HIV in this subgroup of women and consider testing interventions that provide social and/or financial support.

We found a higher risk of HIV among young women who had lived elsewhere for more than three months over the past year, possibly because they are more likely to have had multiple partners or higher-risk partners. A number of studies have provided evidence that mobility is an important risk factor for HIV [4], [11], [36]. Qualitative findings from Mwanza region confirm that young people are often more sexually active when outside their village because of increased opportunity to have sex [29]. Further research should explore whether empowering community members to identify and avoid situations and places where they are more likely to have high risk sex could be an effective risk reduction strategy.

A considerable proportion of HIV prevention efforts to date have focused on encouraging a reduction in the number of sexual partners. This study, as expected, found that the risk of HIV was greater among those with a higher number of lifetime sexual partnerships, and in females was also higher among those with a higher number of partnerships in the year prior to the survey. In this study we attempted to look at the risk associated with potentially riskier sexual partnerships (new partners and concurrent partners). After adjustment for other co-variates, there was evidence of increased risk of HIV both with increasing numbers of new partners and with concurrent partnerships among females. Number of partnerships is a very crude measure of potential risk of infection and future studies of risk factors for HIV should also consider the type of sexual partner, and, if possible the number of (unprotected) sex acts.

The decreased risk of HIV infection among females who had ≥2 lifetime pregnancies is to be expected after adjustment for age, because HIV-infected women have lower fertility [22]. Women with multiple children may also be more likely to be in stable partnerships and have less risky sexual behaviours.

Previous studies have shown that male circumcision provides considerable protection against HIV acquisition [38], [39], [40], [41], [42]. In this study, we did not find evidence of an association between circumcision and HIV, although it is important to note that the confidence intervals on estimates were wide. More detailed analysis of circumcision in this population has demonstrated that those circumcised prior to sexual debut had a 50% lower odds of having HIV, compared to non-circumcised men [38]. The difference in effect measures between these two studies can be explained by a difference in the statistical models used. It is possible that some of the adjustment factors in our model, for example genital ulcers and HSV2, are on the causal pathway from male circumcision to HIV, and that we may therefore have underestimated the “indirect” effects of circumcision on HIV.

The associations observed between HIV infection and having had a blood transfusion in the previous 5 years, higher HIV knowledge, higher condom use, and to a certain extent lower number of lifetime pregnancies, may be due to reverse causality and/or residual confounding. Basic HIV knowledge among young people in these communities was high but not universal. Novel ways to increase knowledge among all community members should be explored such as the use of mobile phones and the internet.

A major strength of the study was the availability of data on a wide range of exposures, including STI status, for a large number (∼14,000) of young people. Another important strength was the use of a conceptual framework within the analysis, which reflected the hierarchical relationship between exposures [20]. Estimation of the effect of variables at a given level was unadjusted for variables at lower levels of the hierarchy, to avoid underestimating effects by controlling for mediating factors that are in the causal pathway. To avoid excessive parameterisation of the model, given the relatively small number of cases of HIV, associations were adjusted only for exposures that remained significant at p<0.10 in the multivariate analysis.

The 20 MEMA kwa Vijana trial communities were broadly representative of rural communities in Mwanza region. During the survey the research team made a number of repeat visits to try to interview young people that they might have missed on earlier visits. Nevertheless, not all those identified during the household census were successfully interviewed so we cannot exclude the possibility of selection bias. Furthermore, only those who had reached at least Standard 5 of primary school were eligible to participate in the survey and so the study population did not include those who did not go to school or who dropped out of school very early. However, under-representation of mobile or less educated members of the community should not have compromised the internal validity of the study.

A further limitation of the study was its focus on individual-level exposures such that the full contribution of some population-level exposures for HIV transmission may not have been captured. As with all cross-sectional studies, an unavoidable limitation was our inability to explore the temporal nature of the associations though many of the exposures identified in this study have previously been identified as risk factors for HIV incidence in different populations in the same study areas.

In conclusion, our results show that risk factors for HIV in this young rural population are complex and interlinked. In the absence of an effective intervention to prevent HSV2, reinforcement of health promotion messages for all community members is essential. This should include education on the increased risk of HIV acquisition associated with certain STIs, and the importance of seeking care for suspected STIs early. Other important risk factors were marital status, mobility, and reported number of sexual partners. A better understanding of types of sexual partnerships and of behaviours within those relationships should lead to more relevant and potentially more effective HIV prevention messages. Furthermore, the possibility that an increase in the number of sexual partnerships or risky behaviour within partnerships occurs when a woman is more vulnerable, such as when a marriage ends or when she is away from her home, suggests that interventions tailored to the needs of these subgroups of women may be useful. Future interventions to reduce HIV infection among rural young people in Mwanza Region are likely to be more successful if they involve the whole community and address broader community-level risk factors.
